# Current Pharmacological Management in Juvenile Huntington’s Disease

**DOI:** 10.1371/currents.RRN1304

**Published:** 2012-03-26

**Authors:** Lisa Robertson, Helen Santini, Kirsty L O'Donovan, Ferdinando Squitieri, Roger A Barker, Maria Rakowicz, G. Bernhard Landwehrmeyer, Oliver Quarrell

**Affiliations:** ^*^Department of Clinical Genetics, Sheffield Children's Hospital, Sheffield UK S10 2TH; ^‡^Neurogenetics and Rare Diseases Centre, IRCCS Neuromed, Pozzilli (IS), Italy.; ^§^University of Cambridge; ^¶^Department of Clinical Neurophysiology, Institute of Psychiatry and Naurology, Warsaw, Poland and ^#^Dept. of Neurology, University of Ulm, Ulm, Germany

## Abstract

Background: The clinical presentation of Juvenile Huntington’s Disease (JHD) can be very different from adult-onset HD with little evidence to guide symptomatic management.

Aim: To survey the current use of pharmacological treatments for JHD.

Methods: Patients were identified through the HD Association, Hospital Doctors and the European Huntington’s Disease Network REGISTRY study.

Results: The most commonly prescribed agents were anti-psychotics (24/45), anti-depressants (17/45) and anti-parkinsonian medications (15/45). 5 patients were taking more than 8 medications.

Conclusions: The most commonly prescribed group of medication was the anti-psychotic. Many patients were on multiple therapies, highlighting the need to rationalise medications.

   Huntington’s disease (HD) is a dominantly inherited, neurodegenerative disorder, due to a (CAG)n repeat expansion in the *HTT* gene[1]. In the majority of HD cases, onset is in adult life.   

Juvenile HD (JHD)is defined as onset under 20 years of age[2] and represents approximately 1-10% of cases[3] [4] [5] [6] [7] .  There is an inverse correlation between the age at onset of disease and the number of (CAG)n repeats [8] [9] [10].   

Adult HD is characterised by a triad of clinical features including: chorea, cognitive decline and psychiatric disturbance or behavioural disturbance [11].  However, in JHD, particularly in those with onset in the first decade, chorea is less likely to be present and instead, patients are more likely to present with rigidity, bradykinesia, dystonia and gait disorder, ie. parkinsonian features [7] [11] [12] [13] [14] [15] [16] [17]. In addition, JHD patients are more likely to develop epilepsy than adult-onset cases[18]. The spectrum of features at presentation can also include ataxia, dysarthria, dysphagia, deterioration in school performance or severe behavioural problems [11] [14] [19] [20]. 

There is currently no effective disease-modifying treatment for either adult onset HD or JHD; therefore, clinical care is symptomatic and supportive. The very different presentation means that different treatments are likely to be used in the management of JHD versus adult onset HD. Given the rarity of the condition, each treating clinician is unlikely to have seen more than a few cases and there are no treatment guidelines. Therefore, gathering together the limited information available is of significant benefit to both families and physicians and helps form the basis of future trials. 

For UK families affected by JHD, we composed a questionnaire, to be completed by the family, listing their child’s symptoms and treatments. The study received M-REC and local R&D approval. Patients were identified through the Patient’s Support Group and Hospital Clinicians (Neurologists and Clinical Geneticists). 

As a second phase, we collated data on medications prescribed for JHD patients enrolled in the European Huntington’s Disease Network REGISTRY study (excluding the UK). We used monitored case report forms from the European Huntington’s Disease Research Network (EHDN) REGISTRY project.  This is a multinational observational study see www.euro-hd.net/html/registry [21]. All medications were grouped according to the WHO ATC classification.  

 In the UK, 40 questionnaires were sent out and seven families responded. 38 REGISTRY patients with JHD were identified.  

In the UK cohort, the most common symptoms reported by the families were speech difficulties(7), dysphagia(6), stiffness/spasticity(6), sleeping difficulty(5), pain(4) and behavioural problems(4) . Equivalent data from REGISTRY was not available. 

When the data was combined the median number of medications prescribed was four, with an arithmetic mean of five (range 1-16) (Fig 1).

**Figure:**
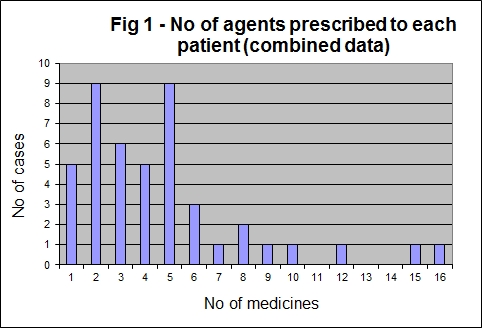
Figure 1: No of agents prescribed to each patient (combined data. 

In REGISTRY, the most commonly prescribed agents were anti-psychotics, anti-depressants, anti-parkinsonianmedications and anti-epileptics (Fig 2). In the UK, the anti-psychotic was the most commonly prescribed agent (Fig 3).

**Figure:**
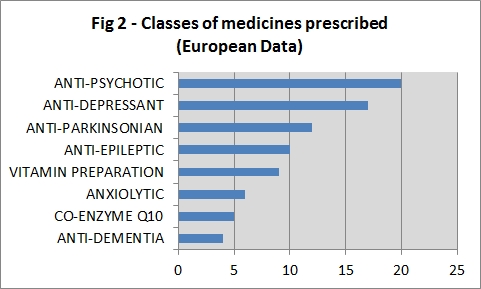
Figure 2: Classes of medicines prescribed (European Data).

**Figure:**
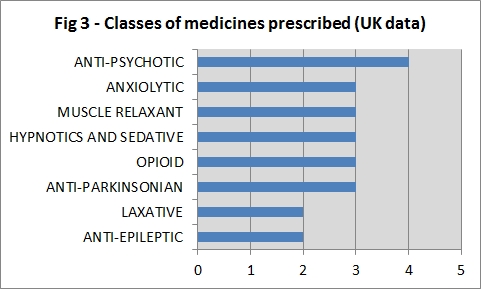
Figure 2: Classes of medicines prescribed (UKData).

The most commonly prescribed individual medication was Valproic acid (anti-epileptic), followed jointly by: Tiapride (anti-psychotic), Dopa and Dopa derivatives (anti-parkinsonian) and Tocopherol (vitamin preparation) (Fig 4).

**Figure:**
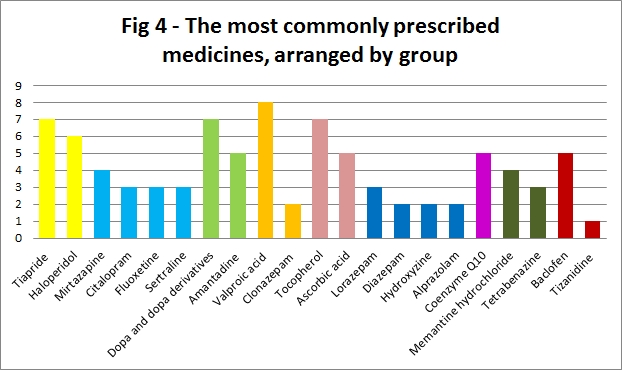
Figure 4: The most commonly prescribed medicines, arranged by group 

## **Discussion                                    ** 

The most commonly prescribed medication in our cohort was the anti-psychotic agent (24/45 individuals).  In the UK group, the family member completing the questionnaire was asked to give the indication for each medication, and for anti-psychotics this included: agitation, behavioural problems and psychiatric disturbances, none of which are listed as diagnostic features of JHD. This is important as it is recognised that children presenting with behavioural or psychiatric problems often experience a significant delay in receiving a diagnosis of JHD [14], and our data would infer that such problems are common in this age group affected by HD.  

Anti-depressants and anti-parkinsonian agents were also frequently prescribed (n=17 and n=15 respectively) and some (n=7) were taking a combination of anti-parkinsonian medications and anti-psychotics. This odd regime often reflects the combination of dystonia and parkinsonism seen in JHD, although whether it is a useful combination is unclear especially given the recognised parkinsonian side-effects of anti-psychotics [22]. This side-effect is less frequent with the atypical anti-psychotics than in first generation anti-psychotics, but does still occur and it may be difficult to distinguish from disease progression.  

The most commonly prescribed individual medication was Valproic acid (8), reflecting consistency in anti-epileptic prescribing. This is in-line with prescribing practice in the management of childhood epilepsy of all causes[23]; Valproic acid is widely used as first-line therapy for generalised seizures with a strong evidence base to support its efficacy[24]. At present, as there is no disease-modifiying therapy available, the  optimum management of JHD encompasses the optimum management of the associated symptoms.

Five patients were prescribed in excess of eight types of medications. The highest number of medications for any one patient was sixteen, raising issues of polypharmacy. This is a particularly important issue in a group of patients with swallowing difficulties.  Three patients were on three or more anti-pyschotic agents or anti-parkinsonism agents simultaneously: which highlights the fact that many cases of JHD appear to be refractory to pharmacotherapies. 

An apparent difference in prescribing habits was noted between the two populations of patients studied: within the UK, opioids were prescribed whereas in the REGISTRY group they were not, this may be due to variations in national restrictions[25]. For certain individuals with JHD, pain can be a feature and from our own experience this can be difficult to manage and the input of a specialist pain team or palliative care specialists can be invaluable[26].  

The diagnostic features of HD presenting in the first decade are a family history of HD, often in the father (though onset in the child may precede onset in the parent), and two or more of the following: declining school performance, seizures, oral motor dysfunction, rigidity and gait disorder [27]. We analysed the data based on age of diagnosis (Fig 4); we hypothesized that those who were older at diagnosis may have a profile more akin to adult JHD with a lower incidence of rigidity, dystonia and epilepsy. We therefore divided patients into three groups, those diagnosed at 10 years of age or under (n=6), those diagnosed between 11 to 15 years of age (n=12) and those diagnosed between 16 to 20 years (n=26). The age at diagnosis was unknown in one case (n=1). We observed that those who were diagnosed at 10 years or under were significantly more likely to be prescribed a muscle relaxant than those diagnosed between ages 11-20 years (Fisher's exact test, p=0.01). There was no apparent trend between younger age at diagnosis and the frequency of prescription of anti-epileptics or anti-parkinsonian agents.  

**Figure:**
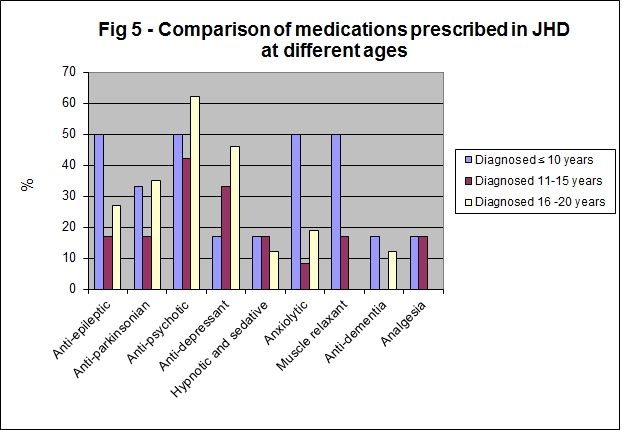
Figure 5: Comparison of medications prescribed in JHD at different ages 

## **Conclusions** 

We set out to identify which medications were being prescribed for symptomatic relief in JHD; this has not been done before. We identified that anti-psychotics were the most commonly prescribed agent and that anti-depressants and anti-parkinsonian medications were also frequently prescribed in the JHD group.     

JHD patients have complex needs. The diversity of their problems means they are often prescribed medications which exceed the area of expertise of a single hospital specialist. Their medications require regular review by a team of experts, in the face of evolving symptoms. Polypharmacy is an issue in this group, highlighting a potential need for reviewing and stopping medicines, especially when they appear to be ineffective. 

Identifying current practice is only a first step towards establishing an evidence base for treatment guidelines. JHD is a rare condition which means that international collaboration is essential to gather information on the effectiveness of symptomatic management. Projects such as REGISTRY are vital in order to establish such information. Ultimately we would hope that it will be possible to use such a database to identify affected patients and conduct a randomised trial on the effectiveness of different symptomatic treatments in this form of HD.   

## **Acknowledgements**

We would like to acknowledge the time and effort of the patients and families who participated in this study.  

We would also like to acknowledge Mrs. Wendy Swan, Clinical Research Co-ordinator, for her assistance with research governance.  

## Funding information

We would like to acknowledge the funding for this project which we received from the Sheffield Children’s Hospital Charity (SCHC). 

## **Competing interests**

The authors have declared that no competing interests exist. 

## References

[ref-1807824456] (1993) A novel gene containing a trinucleotide repeat that is expanded and unstable on Huntington's disease chromosomes. The Huntington's Disease Collaborative Research Group. Cell 72: 971-983. 10.1016/0092-8674(93)90585-e 8458085

[ref-1428774417] Bruyn GW (1969) The Westphal variant and juvenile type of Huntington's chorea. . Progress in neurogenetics International congress series no 175 Amsterdam: Excerpta Medica Foundation 1: 666-673.

[ref-223043794] Harper PS, Morris MR, Quarrell OWJ, Shaw DJ, Tyler AT, et al. (1991) Huntington’s Disease. London: Saunders.

[ref-3604076628] Went LN, Vegter-van der Vlis M, Bruyn GW (1984) Parental transmission in Huntington's disease. Lancet 1: 1100-1102. 10.1016/s0140-6736(84)92510-8 6144830

[ref-3920945579] Groppi C, Barontini F, Bracco L, Sita D, Inzitari D, et al. (1986) Huntington's chorea: a prevalence study in the Florence area. Acta Neurol Scand 74: 266-268. 10.1111/j.1600-0404.1986.tb03512.x 2949477

[ref-4278008593] Oliver JE (1970) Huntington's chorea in Northamptonshire. Br J Psychiatry 116: 241-253. 10.1192/bjp.116.532.241 4244787

[ref-2086892737] Cannella M, Gellera C, Maglione V, Giallonardo P, Cislaghi G, et al. (2004) The gender effect in juvenile Huntington disease patients of Italian origin. Am J Med Genet B Neuropsychiatr Genet 125B: 92-98. 10.1002/ajmg.b.20110 14755452

[ref-1439128938] Claes S, Van Zand K, Legius E, Dom R, Malfroid M, et al. (1995) Correlations between triplet repeat expansion and clinical features in Huntington's disease. Arch Neurol 52: 749-753. 10.1001/archneur.1995.00540320021009 7639626

[ref-362179473] Duyao M, Ambrose C, Myers R, Novelletto A, Persichetti F, et al. (1993) Trinucleotide repeat length instability and age of onset in Huntington's disease. Nat Genet 4: 387-392. 10.1038/ng0893-387 8401587

[ref-2634577125] Persichetti F, Srinidhi J, Kanaley L, Ge P, Myers RH, et al. (1994) Huntington's disease CAG trinucleotide repeats in pathologically confirmed post-mortem brains. Neurobiol Dis 1: 159-166. 10.1006/nbdi.1994.0019 9173995

[ref-3607019573] Nance MA, Myers RH (2001) Juvenile onset Huntington's disease -clinical and research perspectives. Ment Retard Dev Disabil Res Rev 7: 153-157. 10.1002/mrdd.1022 11553930

[ref-2100651727] Nance MA (1997) Genetic testing of children at risk for Huntington's disease. US Huntington Disease Genetic Testing Group. Neurology 49: 1048-1053. 10.1212/wnl.49.4.1048 9339688

[ref-1256550169] Ruocco HH, Lopes-Cendes I, Laurito TL, Li LM, Cendes F (2006) Clinical presentation of juvenile Huntington disease. Arq Neuropsiquiatr 64: 5-9. Epub 2006 Apr 2005. 10.1590/s0004-282x2006000100002 16622544

[ref-2037646442] Ribai P, Nguyen K, Hahn-Barma V, Gourfinkel-An I, Vidailhet M, et al. (2007) Psychiatric and cognitive difficulties as indicators of juvenile huntington disease onset in 29 patients. Arch Neurol 64: 813-819. 10.1001/archneur.64.6.813 17562929

[ref-1533661622] Mahant N, McCusker EA, Byth K, Graham S (2003) Huntington's disease: clinical correlates of disability and progression. Neurology 61: 1085-1092. 10.1212/01.wnl.0000086373.32347.16 14581669

[ref-881035399] Siesling S, Vegter-van der Vlis M, Roos RA (1997) Juvenile Huntington disease in the Netherlands. Pediatr Neurol 17: 37-43. 10.1016/s0887-8994(97)00069-6 9308974

[ref-4177999736] van Dijk JG, van der Velde EA, Roos RA, Bruyn GW (1986) Juvenile Huntington disease. Hum Genet 73: 235-239. 10.1007/BF00401235 2942452

[ref-722950315] Brackenridge CJ (1980) Factors influencing dementia and epilepsy in Huntington's disease of early onset. Acta Neurol Scand 62: 305-311. 10.1111/j.1600-0404.1980.tb03041.x 6451137

[ref-3457907663] Squitieri F, Pustorino G, Cannella M, Toscano A, Maglione V, et al. (2003) Highly disabling cerebellar presentation in Huntington disease. Eur J Neurol 10: 443-444. 10.1046/j.1468-1331.2003.00601.x 12823498

[ref-86224046] Quarrell OW, Brewer HM, Squitieri F, Barker RA, Nance MA, Landwehrmeyer GB (2009) Juvenile Huntington's Disease. Oxford: Oxford University Press.

[ref-3556967130] Orth M, Handley OJ, Schwenke C, Dunnett SB, Craufurd D, et al. (2010) Observing Huntington's Disease: the European Huntington's Disease Network's REGISTRY. PLoS Curr 2.: RRN1184. RRN1184 10.1371/currents.RRN1184 PMC2947793 20890398

[ref-126198696] (2011) British National Formulary.

[ref-2945432100] Wheless JW, Clarke DF, Arzimanoglou A, Carpenter D. Treatment of pediatric epilepsy: European expert opinion, 2007. Epileptic Disord. 2007 Dec;9(4):353-412. Review. 18077226 10.1684/epd.2007.0144

[ref-3383917568] Perucca E, Tomson T. The pharmacological treatment of epilepsy in adults. Lancet Neurol. 2011 May;10(5):446-56. Review. 21511198 10.1016/S1474-4422(11)70047-3

[ref-3123844962] Cherny NI, Baselga J, de Conno F, Radbruch L (2010) Formulary availability and regulatory barriers to accessibility of opioids for cancer pain in Europe: a report from the ESMO/EAPC Opioid Policy Initiative. Ann Oncol 21: 615-626. 10.1093/annonc/mdp581 20176694

[ref-1147639560] King N. Palliative care management of a child with juvenile onset Huntington's disease. Int J Palliat Nurs. 2005 Jun;11(6):278-83. Review. 16010224 10.12968/ijpn.2005.11.6.18295

[ref-2808506138] Rasmussen A, Macias R, Yescas P, Ochoa A, Davila G, et al. (2000) Huntington disease in children: genotype-phenotype correlation. Neuropediatrics 31: 190-194. 10.1055/s-2000-7461 11071143

